# Antiviral Effects of Curcumin on Adenovirus Replication

**DOI:** 10.3390/microorganisms8101524

**Published:** 2020-10-04

**Authors:** Morgan R. Jennings, Robin J. Parks

**Affiliations:** 1 Regenerative Medicine Program, Ottawa Hospital Research Institute, Ottawa, ON K1H 8L6, Canada; mojennings@toh.ca; 2 Department of Biochemistry, Microbiology and Immunology, University of Ottawa, Ottawa, ON K1N 6N5, Canada; 3 Centre for Neuromuscular Disease, University of Ottawa, Ottawa, ON K1N 6N5, Canada; 4 Department of Medicine, The Ottawa Hospital, Ottawa, ON K1H 8L6, Canada

**Keywords:** human adenovirus, antiviral therapy, curcumin

## Abstract

Human adenovirus (HAdV) is a common pathogen that can cause severe morbidity and mortality in certain populations, including pediatric, geriatric, and immunocompromised patients. Unfortunately, there are no approved therapeutics to combat HAdV infections. Curcumin, the primary curcuminoid compound found in turmeric spice, has shown broad activity as an antimicrobial agent, limiting the replication of many different bacteria and viruses. In this study, we evaluated curcumin as an anti-HAdV agent. Treatment of cells in culture with curcumin reduced HAdV replication, gene expression, and virus yield, at concentrations of curcumin that had little effect on cell viability. Thus, curcumin represents a promising class of compounds for further study as potential therapeutics to combat HAdV infection.

## 1. Introduction

Human adenovirus (HAdV) is a non-enveloped, icosahedral, double-stranded DNA (dsDNA) virus, capable of infecting ocular [1], respiratory [2], or gastrointestinal tissues [3]. HAdV is grouped into 7 species (A to G), and further sub-grouped into over 90 different types [2]. In healthy individuals, HAdV infection is typically self-limiting [4]. However, HAdV infection can cause severe morbidity and mortality in certain populations, including pediatric, geriatric, and immunocompromised patients [5]. Currently, there is no therapy specific to HAdV available to the general public, and infections are usually treated with common antiviral drugs, such as cidofovir [6]. Cidofovir, a cytidine analog, inhibits HAdV DNA replication by inhibiting HAdV DNA polymerase, as well as preventing chain elongation when incorporated into the viral DNA [7]. However, cidofovir is often associated with high levels of nephrotoxicity [8,9,10]. Brincidofovir (CMX001, Chimerix Inc., Durham, NC, USA), a lipid conjugate of cidofovir, has improved oral bioavailability and reduced nephrotoxicity [11,12], but has been associated with gastrointestinal toxicity [12]. Immunotherapy using anti-HAdV T-cells has shown some early success in bone marrow transplant patients, but studies are still ongoing and there is a lack of data on the effectiveness of this therapy in solid organ transplant patients [5,12,13]. Additionally, the time-intensive nature of producing anti-HAdV T-cells severely limits the wider adoptability of this therapy [12].

An effective method used to discover novel antiviral compounds is to screen libraries of small molecules to assess their effect on viral replication. Several groups have performed such high throughput screens (HTS) to identify compounds affecting HAdV [6,14,15,16,17]. Using reporter viruses derived from several HAdV types and expressing green fluorescent protein (GFP), Sanchez-Cespedes et al. [14] identified the piperazinone derivative 15D8 as an effective inhibitor of HAdV replication. Using an assay based on inhibition of cytopathic effect in cells, Hartline et al. [15] performed a screen of 16 compounds for efficacy against several different DNA viruses, including HAdV, and identified filociclovir, a nucleoside analogue [18], as effective against HAdV and several other viruses.

Our research group developed an HTS protocol to identify compounds that inhibit HAdV replication [16]. We utilized a HAdV type 5-based construct that expressed red fluorescent protein (RFP) as part of the late transcription unit, such that RFP is only expressed at appreciable levels following viral DNA replication [19]. As such, the degree to which a test compound affects viral gene expression and replication inversely correlates with quantity of RFP present in the treated cells [16]. Using this HTS strategy, we tested the Prestwick library (~1200 compounds, most of which are FDA-approved) for compounds affecting HAdV infection and identified 11 compounds with anti-HAdV activity [16]. Follow-up studies on three cardiotonic steroids (digoxin, digitoxigenin, and lanatoside C) identified in this screen showed that these compounds primarily affected early 1 A (E1A) expression, and ultimately all reduced virus yield from treated cells [16]. We also screened the Cayman Epigenetic Screening library, containing 150 small molecules that modulate the activity of epigenetic regulatory proteins, including methyltransferases, demethylases, histone acetyltransferases and deacetylases, and acetylated lysine readers, and identified 19 compounds exhibiting anti-HAdV activity [17]. Finally, we showed that suberoylanilide hydroxamic acid (SAHA), a histone deacetylase (HDAC) inhibitor, effectively inhibits HAdV replication at several stages in the HAdV lifecycle, including gene expression and DNA replication [19]. The effect of SAHA was attributed to inhibition of Class I HDAC, primarily HDAC2, showing that HDAC activity is required for normal HAdV replication. Thus, compounds identified in these screens may act as effective anti-HAdV therapeutics in addition to providing insight into basic virus biology.

A promising class of compounds that show broad antimicrobial activity are the curcuminoids. Curcumin (diferuloylmethane) is a polyphenolic chemical naturally produced by the turmeric plant (*Curcuma longa*), and is the primary curcuminoid compound found in turmeric spice [20]. Curcumin has a variety of biological activities, impacting many different cellular pathways [21,22]. Curcumin has shown efficacy in a variety of model systems, including models of cancer [23,24,25], inflammation [26] and wound healing [27]. Curcumin also exhibits anti-bacterial [28] and anti-viral properties (reviewed in Reference [29]). Indeed, curcumin has shown wide-ranging antiviral activity against many diverse viral species, including single-stranded RNA (ssRNA) and dsDNA viruses [29]. Mechanistically, curcumin can exert antiviral effects either directly on virus-encoded factors [30,31,32], or through affecting cellular processes or pathways crucial for normal virus function [33,34]. In this study, we investigated the efficacy of curcumin as an anti-HAdV compound.

## 2. Materials and Methods

### 2.1. Cell lines, Viruses and Reagents

Experiments were conducted in the human lung adenocarcinoma-derived A549 cell line (CCL-185, American Type Culture Collection (ATCC), Manassas, VA, USA), unless stated otherwise. Cells were cultured in Minimum Essential Medium (MEM, Sigma Aldrich, St. Louis, MO, USA) containing 10% (*v/v*) Fetal Bovine Serum (FBS, Sigma Aldrich), 2 mM GlutaMAX (Invitrogen, Carlsbad, CA, USA), and 1x antibiotic-antimycotic (Invitrogen). HAdV-4 (VR-4), and HAdV-7 (VR-7) were obtained from the ATCC, and stocks were propagated and titered on A549 cells. HAdV-5 was obtained from Dr. John Bell (Ottawa Hospital Research Institute, Ottawa, Canada), and it was grown and titered on 293 cells.

**Figure 1:**
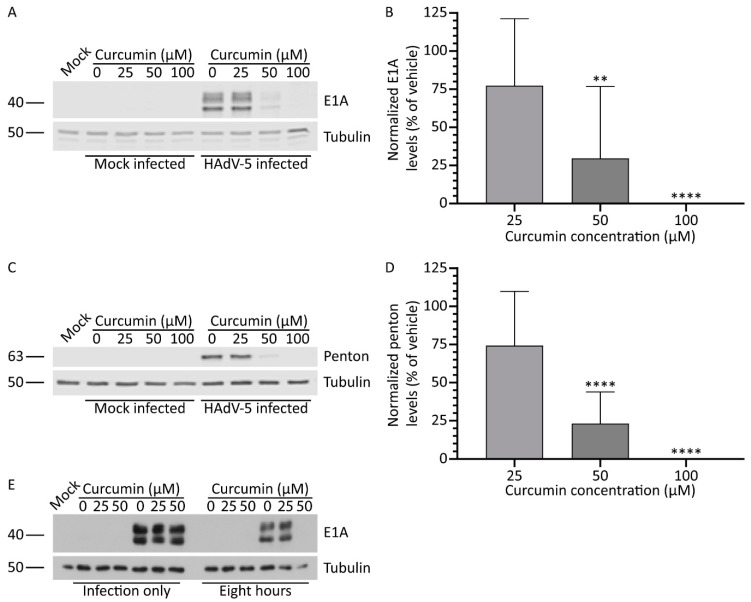
Treatment with curcumin reduces human adenovirus (HAdV)-5 protein levels. A549 cells were infected with HAdV-5 at a multiplicity of infection (MOI) of 10 for 1 h, and overlaid with medium containing curcumin (0, 25, 50, and 100 μM). At 8 and 24 hours post-infection (hpi), crude cell protein extracts were collected for immunoblot analysis. (**A**,**B**) Samples prepared at 8 hpi were analyzed for quantity of the early 1 A (E1A) protein. (**C**,**D**) Samples harvested 24 hpi were probed with antibody to HAdV-5 capsids proteins. Images and quantification for penton protein are shown. Signal intensities were quantified using Odyssey CLx imaging system, with each sample normalized to the tubulin loading control. Values are plotted relative to vehicle-treated cells. The mean of nine experiments are shown and the error bars represent standard deviation (SD) of the mean. (**E**) A549 cells were infected with HAdV-5 at an MOI of 10 for 1 h in the presence of 0, 25, and 50 μM of curcumin. One hpi, one group received medium containing no curcumin, while the other received medium containing the indicated concentrations of curcumin. At 8 hpi, crude cell protein extracts were prepared and analyzed by immunoblot for E1A, with tubulin as a loading control. ** *p* ≤ 0.01, **** *p* ≤ 0.0001.

**Figure 2:**
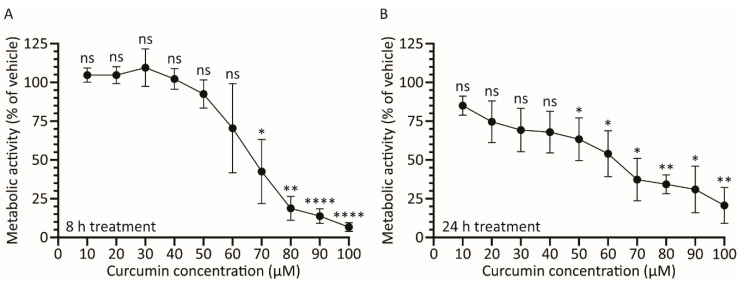
Treatment with curcumin causes a dose-depended decrease in cellular metabolic activity. A549 cells in 96-well plates were incubated with medium containing curcumin from 0–100 μM. Cellular metabolic activity was determined 8 (**A**) or 24 (**B**) h later. The mean of three experiments are shown and the error bars represent standard deviation (SD) of the mean. * *p* ≤ 0.05, ** *p* ≤ 0.01, **** *p* ≤ 0.0001, ns *p* > 0.05.

**Figure 3:**
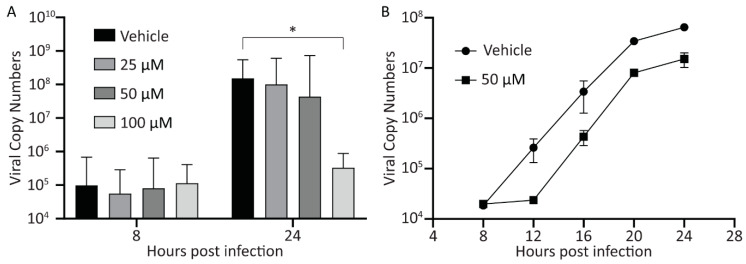
Treatment with curcumin reduces HAdV-5 genome copy number within cells. (**A**) A549 cells were infected with HAdV-5 at an MOI of 10 and overlaid with medium containing curcumin (0, 25, 50, and 100 μM). At 8 and 24 hpi, total DNA was isolated from the cells by sodium dodecyl sulphate (SDS)-proteinase K digestion and phenol/chloroform extraction. The resulting DNA was subjected to qPCR to determine the average genome copy number per 200 ng DNA, normalized to the average copy number of human GAPDH. The mean of three experiments are shown and the error bars represent standard deviation (SD) of the mean. (**B**) A549 cells were infected with HAdV-5 at an MOI of 10 and overlaid with medium containing curcumin (0 and 50 μM). Eight hpi and every 4 h until 24 hpi, total DNA was isolated from the cells by SDS-proteinase K digestion and phenol/chloroform extraction. The resulting DNA was subjected to qPCR to determine the average genome copy number per 200 ng DNA, normalized by average copy number of human GAPDH. Data represents a single experiment analyzed in duplicate, with error bars representing standard deviation (SD) of the mean. * *p* ≤ 0.05.

**Figure 4:**
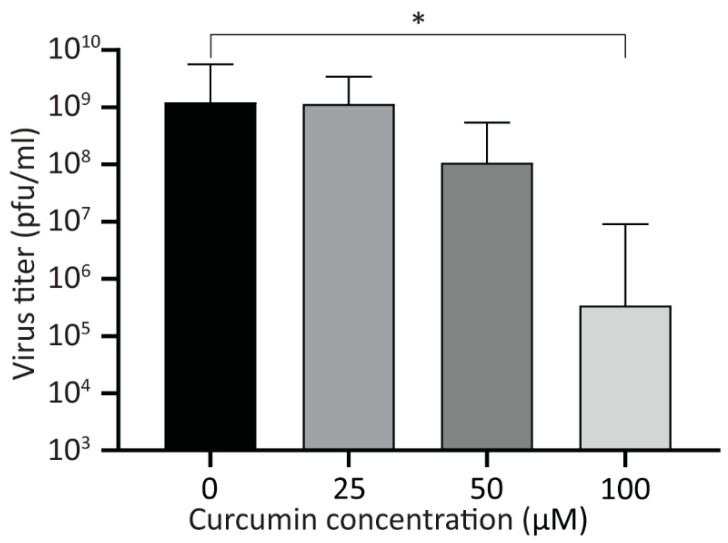
Treatment with curcumin reduces viral yield. A549 cells were infected with HAdV-5 at an MOI of 10 for 1 h and overlaid with medium containing curcumin (0, 25, 50, and 100 μM). Twenty-four hpi, the cells were harvested into the medium, and the titer of recovered viruses was analyzed by plaque assay. The mean of three experiments are shown, and the error bars represent standard deviation (SD) of the mean. * *p* ≤ 0.05.

**Figure 5:**
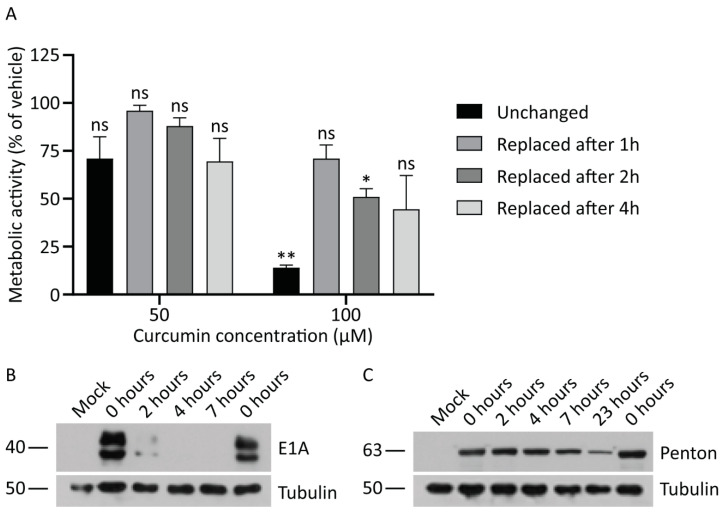
Continued exposure to curcumin is required to inhibit HAdV replication. (**A**) A549 cells were treated with medium containing 0, 50, or 100 μM of curcumin. Medium was removed from the plates after 1, 2, or 4 h and replaced with fresh medium for the remainder of the 24-h time course. As a control, a series of plates were incubated in the presence of curcumin for the entire 24 h. Cellular metabolic activity was determined at the 24-h timepoint by MTS assay. The mean of two experiments are shown and the error bars represent standard deviation (SD) of the mean. (**B**,**C**) A549 cells were infected with HAdV-5 at an MOI of 10 for 1 h and overlaid with medium containing curcumin (50 μM) or vehicle. After 2, 4, 7, and 23 h incubation in the presence of curcumin, the medium was replaced with fresh medium lacking curcumin. Eight and 24 hpi, crude cell protein extracts were prepared and analyzed by immunoblot for E1A and late protein levels, with tubulin as a loading control. * *p* ≤ 0.05, ** *p* ≤ 0.01, ns *p* > 0.05.

**Figure 6:**
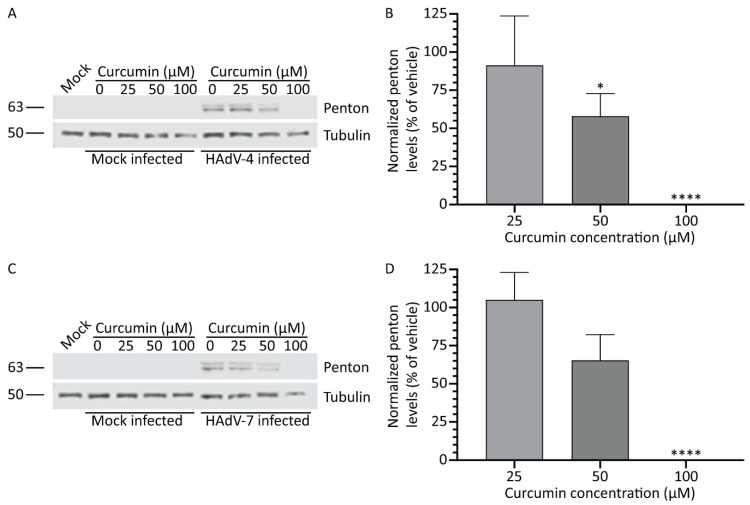
Treatment with curcumin reduces HAdV types 4 and 7 protein levels. A549 cells were infected with HAdV-4 or -7 at an MOI of 10 for 1 h, and overlaid with medium containing curcumin (0, 25, 50, and 100 μM). Twenty-four hpi, crude cell protein extracts were prepared and analyzed by immunoblot for late protein levels. (**A**) Samples infected with HAdV-4 were analyzed for expression of several late HAdV proteins by immunoblot using an α-HAdV-5 antibody. (**B**) Quantification of penton protein from HAdV-4 (~63 kDa major band) normalized to tubulin. Values are presented relative to vehicle-treated cells. (**C**) Samples infected with HAdV-7 were analyzed for expression of several late HAdV proteins by immunoblot using an α-HAdV-5 antibody. (**D**) Quantification of penton protein from HAdV-7 (~63 kDa major band) normalized to tubulin. Values are presented relative to vehicle-treated cells. The mean of three experiments are shown, and the error bars represent standard deviation (SD) of the mean. * *p* ≤ 0.05, **** *p* ≤ 0.0001.

**Figure 7:**
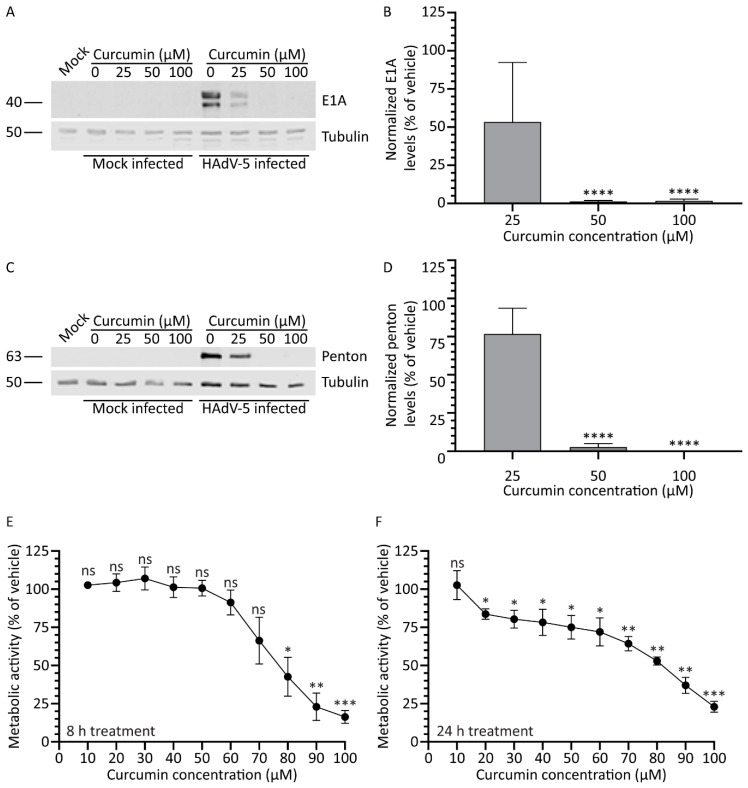
Treatment with higher purity curcumin improves efficacy against HAdV. A549 cells were infected with HAdV-5 at an MOI of 10 for 1 h and overlaid with medium containing curcumin of higher purity (0, 25, 50, and 100 μM). (**A**,**B**) Samples prepared at 8 hpi were analyzed for the quantity of the early protein E1A. (**C**,**D**) Samples harvested 24 hpi were probed with antibody to HAdV-5 capsid proteins. Images and quantification for penton protein are shown. Signal intensities were quantified using Odyssey CLx imaging system, with each sample normalized to the tubulin loading control. (**E**,**F**) A549 cells in 96-well plates were incubated with medium containing higher-purity curcumin from 0–100 μM. Cellular metabolic activity was determined 8 (**E**) or 24 (**F**) h later. Values are plotted relative to vehicle-treated cells. The mean of three experiments are shown, and the error bars represent standard deviation (SD) of the mean. * *p* ≤ 0.05, ** *p* ≤ 0.01, *** *p* ≤ 0.001, **** *p* ≤ 0.0001, ns *p* > 0.05.

The curcumin used in the experiments described in Figure 1, Figure 2, Figure 3, Figure 4, Figure 5 and Figure 6 was obtained from Sigma Aldrich (≥65% purity, C1386), while the curcumin used in the experiments described in Figure 7 was obtained from Cayman Chemical (≥90% purity, 81025, Ann Arbor, MI, USA). Curcumin was freshly dissolved in dimethyl sulfoxide (DMSO, BP231-1, Thermo Fisher Scientific, Waltham, MA, USA) to prepare a stock solution before each experiment. The stock solution of curcumin was diluted to the desired concentration in cell medium with a final DMSO concentration of 0.25% for each treatment.

### 2.2. Infection and Drug Treatments

Medium was removed from confluent monolayers of A549 cells prior to infection with HAdV in a minimum volume. The multiplicity of infection (MOI) was calculated as plaque-forming units (PFU) per cell, and an MOI of 10 was used for all experiments, unless specified otherwise. Virus inoculums were diluted in phosphate-buffered saline (PBS, Sigma Aldrich), and added to the cells for 1 hour (h) at 37 °C with periodic rocking. Medium containing either vehicle or curcumin was then added to the cells, followed by incubation in a humidified CO_2_ incubator at 37 °C until the indicated time points. Unless otherwise noted, the indicated hours post-infection (hpi) are from the initiation of infection. Thus, for example, at 8 hpi, the infected cells would have been exposed to curcumin for 7 h.

### 2.3. Immunoblot Analysis

At the indicated timepoints, medium was removed, and the cells were lysed in 2x Laemmli buffer (62.5 mM Tris-HCl pH 6.8, 25% *w/v* glycerol, 2% *w/v* sodium dodecyl sulphate (SDS), 0.01% *w/v* bromophenol blue) containing 5% *v/v* β-mercaptoethanol, and stored at −20 °C. Samples were boiled for 5 min prior to protein separation by sodium dodecyl sulphate-polyacrylamide gel electrophoresis (SDS-PAGE). Separated proteins were transferred to an Immobilon-P polyvinylidene difluoride (PVDF) membrane (Millipore, Burlington, MA, USA). The membrane was blocked with 5% *w/v* skim milk powder dissolved in Tris-buffered saline (20 mM Tris-HCl pH 7.6, 135 mM NaCl) containing 0.2% Tween 20 (Thermo Fisher Scientific) (TBST) and probed with antibodies diluted in the 5% milk solution. The following primary antibodies were used: HAdV-5 E1A (1/5000 typically incubated overnight; MA5-13643, Invitrogen), mouse tubulin (1/10,000 for 1 h incubation; CP06, Millipore), rabbit tubulin (1/10,000 for 1 h incubation; ab59680, Abcam, Cambridge, UK), anti-Adenovirus Type 5 (α-HAdV-5, 1/10,000 for 1 h incubation; ab6982, Abcam). The membranes were then washed three times in TBST and incubated with the appropriate secondary antibodies conjugated to horseradish peroxidase (HRP, BioRad, Hercules, CA, USA). Blots were developed using the Immobilon Classico Western HRP Substrate (Millipore) and visualized by standard autoradiography. All immunoblot data are representative of three or more independent experiments.

To more accurately quantify protein band intensities, immunoblots were processed for and analyzed using an Odyssey CLx imaging system (Li-Cor Biosciences, Lincoln, NE, USA). Separated proteins were transferred to an Immobilon-FL PVDF membrane (Millipore), and the membrane was blocked with Intercept Blocking Buffer (Li-Cor Biosciences). The following primary antibodies were used: HAdV-5 E1A (1/5000 incubated overnight), mouse tubulin (1/10,000 for 1 h incubation), rabbit tubulin (1/10,000 for 1 h incubation), and α-HAdV-5 (1/10,000 for 1 h incubation), which were diluted in Intercept Blocking Buffer solution containing 0.2% Tween 20. The membrane was then washed three times in PBS containing 0.2% Tween (PBST) and incubated with the appropriate IRDye secondary antibodies (680RD and 800CW, Li-Cor Biosciences), diluted in Intercept Blocking Buffer solution containing 0.2% Tween 20 and 0.01% SDS and protected from light. The membrane was washed three times in PBST while still protected from light, followed by a final rinse with PBS. Membranes were then scanned using an Odyssey CLx system (Li-Cor Biosciences) and analyzed using Image Studio Lite (version 5, Li-Cor Biosciences). All protein quantification data are representative of three or more independent experiments.

### 2.4. Quantitative Real-Time PCR (qPCR)

A549 cells were infected and incubated in medium containing curcumin as described above. At the indicated time points, medium was removed, and the cells were harvested using SDS-proteinase K (10 mM Tris-HCl pH 7.4, 10 mM EDTA, 1% *w/v* SDS, 1 mg/mL proteinase K) and incubated overnight at 37 °C. DNA was extracted from the cell lysates using a standard phenol-chloroform method, precipitated with ethanol and NaCl, and the resulting DNA pellet was dissolved in 1x Tris-EDTA (TE). qPCR was performed using 200 ng of genomic DNA per reaction. The following primers were used: 5’-CTC CCC ACA CAC ATG CAC TTA and 5’-CCT AGT CCC AGG GCT TTG ATT for human glyceraldehyde-3-phosphate dehydrogenase (GAPDH); 5’-CCA TTA AAC CAG TTG CCG TGA GAG and 5’-GGC GTT TAC AGC TCA AGT CCA AAG for HAdV E1A. Viral genome copy numbers were calculated from the Ct values using a standard curve obtained using serial dilutions of pCB6, a bacterial plasmid containing the entire HAdV-5 genome. Values were normalized using GAPDH copy numbers, calculated from a standard curve obtained using serial dilutions of a bacterial plasmid containing a cloned fragment of the human GAPDH gene, designated pMJ100. To generate pMJ100, a 99 bp fragment of the human GAPDH gene (generated using PCR primers 5’-CTC CCC ACA CAC ATG CAC TTA and 5’-CCT AGT CCC AGG GCT TTG ATT) was cloned into SmaI-digested pBlueScript II KS(+), verified by sequencing, and purified by cesium chloride buoyant density centrifugation.

### 2.5. Plaque Assay for Virus Yield

To determine the effect of curcumin on virus yield, we performed a plaque assay of virus recovered from curcumin-treated cells. Briefly, monolayers of A549 cells were infected with HAdV-5 at an MOI of 10. The virus inoculum was removed after one h of infection, cells were washed with PBS to remove unbound virus, and fresh medium containing vehicle or curcumin was added. After 24 h of infection, the cells were collected by scraping into the medium, 40% *w/v* sucrose (diluted in 10 mM Tris pH 8.0) was added to a final concentration of 4% *w/v*, and the samples lysed by three freeze/thaw cycles. For the plaque assay, monolayers of A549 cells were infected with dilutions of the cell lysates. After 1 h of infection, the cells were overlaid with medium containing agarose (50% *v/v* of a 1% *w/v* agarose solution, 43% clear 2x MEM, 5% FBS, 1% GlutaMAX, and 1% antibiotic-antimycotic). Plaques were counted 10 days later.

### 2.6. MTS Metabolic Activity Assays

For the metabolic activity assays, 96-well plates were seeded with 5000 A549 cells per well and incubated overnight. The next day, the medium was removed from the wells and fresh medium containing either vehicle or curcumin was added, and incubated for the required time. Metabolic activity was determined using the CellTiter 96 Aqueous Non-Radioactive Cell Proliferation Assay (Promega, Madison, WI, USA) according to the manufacturer’s instructions. Briefly, cells were incubated for 1 h at 37 °C with 20 µL of the 3-(4,5-dimethylthiazol-2-yl)-5-(3-carboxymethoxyphenyl)-2-(4-sulfophenyl)-2H-tetrazolium (MTS) substrate, and absorbance readings were obtained at 490 nm using the SpectraMax 190 plate spectrophotometer (Molecular Devices, San Jose, CA, USA). As curcumin can alter the color of the medium, all absorbances were corrected using wells containing curcumin-treated medium but lacking cells.

### 2.7. Statistical Analysis

Statistical analysis was performed using GraphPad Prism8 software (version 8, GraphPad Software Inc., San Diego, CA, USA). A two-tailed unpaired *t*-test with Welch’s correction was used for comparing treatment to mock or vehicle. Differences with *p* ≤ 0.05 were considered significant.

## 3. Results

### 3.1. Treatment with Curcumin Reduces HAdV-5 Protein Expression

Curcumin has broad anti-viral activity and can inhibit replication of a diverse group of viruses [29]. To investigate whether curcumin can inhibit HAdV replication, we first examined early and late protein expression from the virus in cells treated with varying concentrations of curcumin. A549 cells were infected with HAdV-5 at an MOI of 10 and, 1 hpi, medium containing curcumin (0, 25, 50, and 100 μM) was added. An MOI of 10 was chosen to ensure sufficient E1A production for detection by immunoblot analysis. At 8 and 24 hpi, medium was removed and the cells were collected in 2x Laemmli buffer. For the samples collected at the early 8hpi time point, we examined the quantity of E1A protein within the cells, as the E1A region is the first region to be transcribed following entry of viral DNA into the nucleus, and the E1A species of proteins are vital for stimulating subsequent aspects of the viral replicative cycle [35]. Treatment with curcumin caused a dose-dependent decrease in the quantity of E1A protein in the HAdV-infected cells. At 8 hpi, a trend appeared toward lower E1A protein in cells treated with 25 μM curcumin, which reached significance at 50 μM, and was below the level of detection at 100 μM (Figure 1A). E1A expression was quantified using the Odyssey CLx imaging system, with E1A levels reduced to 75%, 30%, and 0% of vehicle with 25, 50, and 100 μM of curcumin, respectively (Figure 1B). Samples harvested 24 hpi were also analyzed by immunoblot using the Odyssey imaging system, using an α-HAdV-5 antibody capable of binding to several late HAdV-5 proteins, such as the capsid proteins hexon, penton, and fiber. As observed for E1A protein, there appeared a dose-dependent decrease in the quantity of late proteins present in the curcumin-treated cells (Figure 1C). The decrease in late proteins reached significance at concentrations 50 μM and above, as shown by quantification of penton protein levels within the infected cells (Figure 1D). Of note, treatment with 50 mM of curcumin led to an identical level of inhibition of late gene expression when the experiment was repeated with HAdV at an MOI of 1, 5, or 10. Thus, treatment with curcumin reduced the quantity of both early and late viral proteins in HAdV-5-infected cells.

Curcumin can exert its anti-viral effects through multiple mechanisms, including preventing the virus from entering the cell. Curcumin can directly inactivate the virion prior to infection [36], sterically interfere with cellular receptor engagement [37], or inhibit cellular pathways necessary for internalization [34]. Indeed, curcumin was shown to suppress PI3K/Akt signalling [38], which is required for HAdV internalization [39]. Thus, the curcumin-induced reduction in HAdV gene expression may be due to reduced virus entry. To test this possibility, A549 cells were infected with HAdV-5 at an MOI of 10 in the presence of curcumin (0, 25, and 50 μM), and 1 hpi, one group received medium lacking curcumin, while the other received medium containing curcumin (0, 25, and 50 μM). At 8 hpi, medium was removed and the cells were collected in 2x Laemmli buffer. Exposure of the infected cells to 50 μM curcumin for the entire 8 h of infection prevented detectable expression of E1A (Figure 1E). However, treatment with curcumin during only the 1 h of infection did not lower E1A levels, indicating curcumin does not inactivate the virus or prevent internalization, at least at the concentrations tested.

### 3.2. Treatment with Curcumin Causes a Dose-Dependent Decrease in A459 Cellular Metabolic Activity

The effect of curcumin on HAdV protein expression could be due to a direct effect of the drug on the virus, or indirect due to the effect of the drug on host cell health. We thus examined the effect of curcumin on A549 cell metabolic activity. Briefly, A549 cells in a 96-well plate were treated with medium containing 0–100 μM curcumin and, 8 and 24 h later, and assayed for cellular metabolic activity using the CellTiter 96 Aqueous Non-Radioactive Cell Proliferation assay. After 8 h of incubation with curcumin, concentrations of 50 μM and below showed no significant difference in metabolic activity relative to vehicle, although higher concentrations of curcumin adversely affected cell metabolism (Figure 2A). Thus, at 8 hpi, the 75% reduction in E1A protein we observed in HAdV-infected cells treated with 50 μM curcumin (Figure 1A,B) is likely due to direct effects of the drug on the virus and not due to indirect effects on host cell health. Conversely, the complete loss of E1A protein levels we observe at 100 μM of curcumin is due to adverse effects on host cell health. At the 24 h timepoint, cells treated with 50 μM of curcumin showed a significant ~30% reduction in metabolic activity, suggesting that reduced penton levels observed in treated cells (Figure 1C,D) may be due, at least in part, to effects on host cell health rather than solely due to direct effects of curcumin on HAdV function.

### 3.3. Treatment with Curcumin Reduces HAdV-5 Genome Copy Number within Cells

Given that treatment with curcumin can reduce the quantity of both viral early and late proteins within cells, we next examined whether genome copy number of the virus was also reduced. A549 cells were infected with HAdV-5 at an MOI of 10 for 1 h, and incubated in curcumin-containing medium until 8 or 24 hpi. DNA isolated from the infected cells was subjected to qPCR with primers to an amplicon located within the viral E1A region and also the cellular gene GAPDH. All cells showed a similar viral genome copy number at 8 hpi (Figure 3A). At 24 hpi, we observed a ~2-fold and ~5-fold decline in genome copy number in cells treated with 25 and 50 µM of curcumin, respectively, although these differences did not reach significance. However, cells treated with 100 µM exhibited almost a 3-log reduction in viral genome copy number, as expected based on the significant effect curcumin has on cell health at this concentration (Figure 2B). Indeed, the quantity of viral genome present in cells treated with 100 µM of curcumin was not significantly different from samples analyzed at 8 h.

We also examined the kinetics of viral DNA replication in the presence of 50 µM of curcumin. A549 cells were infected with HAdV-5 at an MOI of 10 for 1 h, and incubated in curcumin-containing medium. DNA was isolated from infected cells at 8 hpi, and then every subsequent 4 until 24 hpi. Isolated DNA was subjected to qPCR using the same primers as above. Treatment of cells with curcumin appeared to delay the onset of viral DNA replication by 4 h (Figure 3B). However, once viral DNA replication had initiated, the rate of replication appeared similar between curcumin- and vehicle-treated cells, although the peak quantity of viral DNA at 24 hpi was reduced by ~5-fold in the curcumin treated cells, similar to the previous experiment (Figure 3A). Therefore, treatment with curcumin causes a delay in the onset of HAdV DNA replication.

### 3.4. Treatment with Curcumin Reduces Viral Yield

Next, we examined the effect of curcumin on virus yield. A549 cells were infected with HAdV-5 at an MOI of 10, and 1 hpi, the cells were washed extensively to remove unattached virus, and medium containing curcumin (0, 25, 50, and 100 μM) was added. At 24 hpi, the infected cells were collected into the medium, and the recovered virus was analyzed by plaque assay. While 50 μM of curcumin lowered virus yield by appoximately one log, this was not statistically significant (Figure 4). However, 100 μM of curcumin significantly reduced viral yield by approximately 3.5 log. Since there are no detectable early or late viral proteins in HAdV-infected cells treated with 100 μM curcumin (Figure 1), and no significant increase in viral genome copy number within the cell (Figure 3A), the virus present at 24 hpi in 100 μM curcumin-treated cells likely represents residual virus from the infecting inoculum, as we have observed previously [40]. Indeed, there was no difference in virus recovery when comparing the titer of virus recovered at 4 hpi (before virus DNA replication) for vehicle or 100 μM of curcumin treated cells with that of virus recovered at 24 hpi in the cells treated with 100 μM curcumin. Thus, treatment of cells with curcumin causes a reduction in early and late proteins within the HAdV-infected cell, ultimately reducing virus yield.

### 3.5. Continued Exposure to Curcumin is Required to Inhibit HAdV Protein Expression

Our data indicates that the concentrations of curcumin that abrogate HAdV infection are in the range that can have significant adverse effects on cell function. Given this narrow therapeutic window, we asked whether transient exposure to curcumin could inhibit HAdV replication while preserving cell function. We first examined cell metabolic activity in cells exposed to either 50 or 100 μM of curcumin for different periods of time. Briefly, A549 cells in a 96-well plate were treated with medium containing 0, 50, or 100 μM of curcumin for either 1, 2, or 4 h, at which point the cells were washed and fresh medium without curcumin was replaced, or the cells were exposed to curcumin for the entire 24-h period. Metabolic activity in all cells was examined after 24 h. As shown in Figure 5A, incubation of cells with 50 μM curcumin for all time periods caused a similar minor reduction in metabolic activity which was not significantly different from cells treated with vehicle. For cells treated with 100 μM curcumin, exposure to the drug for the entire 24 h period caused a significant ~90% reduction in metabolic activity. However, treatment with 100 μM curcumin for 1, 2, or 4 h preserved metabolic activity, although there was still a trend toward reduced activity relative to cells treated with vehicle. Thus, there are conditions under which cells can be treated with higher doses of curcumin, and metabolic activity can be preserved.

We next asked whether transient treatment with curcumin was sufficient to abrogate HAdV replication. A549 cells were infected with HAdV-5 at an MOI of 10 for 1 h, at which point medium containing 50 μM of curcumin was added. The cells were incubated in the presence of the drug for 2, 4, 7, and 23 h (i.e., equivalent to 3, 5, 8, and 24 hpi, respectively), washed with PBS and fresh medium with no curcumin replaced. In addition, a control plate of HAdV-infected cells received medium supplemented with vehicle for the entire 8 or 24 h period. At 8 and 24 hpi, crude protein lysates were collected and analyzed by immunoblot for early and late proteins. Treatment with 50 μM of curcumin prevented expression of E1A protein at the 8 hpi when the cells were exposed to drug for the first 4 or more h of infection; however, removal of the drug after 2 h led to detectable levels of E1A protein at the 8 h time point (Figure 5B). This observation suggests that continued exposure to curcumin is required for anti-HAdV efficacy. Indeed, for late protein expression, we observed an inverse correlation between time of exposure to drug and quantity of penton present within the infected cells (Figure 5C). Thus, removal of curcumin allows the virus to initiate gene expression, albeit with delayed kinetics that is dependent on the length of exposure, indicating that the cells need constant exposure to curcumin in order to effectively limit HAdV protein expression.

### 3.6. Treatment with Curcumin Reduces HAdV Types 4 and 7 Protein Levels

Our study shows that curcumin can limit HAdV-5 gene expression and replication. However, HAdV-5 is not the most prevalent serotype associated with human disease and accounts for less than 4% of all HAdV infection cases reported in the USA [41]. HAdV-4 and HAdV-7 are typically associated with more severe disease, accounting for 12.4 and 8.5%, respectively, of all reported cases in patients [41]. Therefore, we next examined whether curcumin could be used to control infection by other, more clinically relevant HAdV types. A549 cells were infected with either HAdV-4 or HAdV-7, treated with medium containing curcumin (0, 25, 50, and 100 μM), and the cells were harvested 24 hpi in 2x Laemmli buffer. We examined late protein expression for these viruses using the α-HAdV-5 antibody, as several of the HAdV-4 and HAdV-7 capsid proteins cross-react with these antibodies [16]. Similar to HAdV-5, we observed a dose-dependent reduction in late proteins in samples infected with HAdV-4 and HAdV-7 and treated with 50 and 100 μM of curcumin (Figure 6A,C). The quantity of penton capsid protein (~65 kDa major band) within the infected cells was significantly reduced for HAdV-4 at concentrations of curcumin 50 μM and above, while HAdV-7 showed a statistically significant reduction in late protein levels only at 100 μM of curcumin (Figure 6B,D). Thus, curcumin is capable of reducing HAdV protein expression in all three types of HAdV tested.

### 3.7. Treatment with Curcumin of Higher Purity Improves Efficacy and Selectivity against HAdV

The curcumin used in our study to this point was reported ≥65% pure. Thus, there may be a significant level of impurities in our curcumin preparations which may affect efficacy of the test compound. We obtained curcumin of greater purity (≥90% pure) and examined its effect on HAdV gene expression and cell health. Briefly, A549 cells were infected with HAdV-5 at an MOI of 10 and, 1 hpi, medium containing the higher purity curcumin (0, 25, 50, and 100 μM) was added. Samples were harvested 8 and 24 hpi for immunoblot analysis. Similar to our previous results, treatment with curcumin caused a dose-dependent decrease in the quantity of E1A protein in the HAdV-infected cells (Figure 7). However, perhaps unsurprisingly, treatment with curcumin of higher purity appeared to have a greater effect on viral gene expression compared to curcumin of lower purity: less E1A protein was present within the infected cells at 25 μM for the higher-purity curcumin relative to less pure curcumin (Figure 1A,B). E1A protein was undetectable in cells treated with 50 μM of the high purity curcumin (Figure 7A). Quantification of signal intensities showed that E1A protein levels were reduced to 53%, 1%, and 1% of vehicle with 25, 50, and 100 μM of curcumin, respectively (Figure 7B). As observed for E1A protein, a dose-dependent decrease appeared in the quantity of late proteins present in the curcumin-treated cells. Indeed, treatment with 50 μM or above of the higher-purity curcumin resulted in undetectable levels of penton (Figure 7C,D).

We additionally analyzed the effect of the higher-purity curcumin on cell health. Briefly, A549 cells were treated with medium containing 0–100 μM higher-purity curcumin and, 8 and 24 h later, assayed for cellular metabolic activity. Compared to the lower purity curcumin, the higher purity curcumin was slightly less toxic to the cells. An 8 h treatment with the higher-purity curcumin had no effect on cellular metabolic activity until concentrations exceeded approximately 70 μM (Figure 7E). Treatment of cells with the higher-purity curcumin resulted in no greater than a ~25% reduction in cellular metabolic activity up to a concentration of 60 μM (Figure 7E,F); however, concentrations above this had a greater deleterious effect on cell health. Thus, treatment of cells with higher-purity curcumin appears to improve the efficacy of the compound against HAdV with no additional increase in cellular toxicity.

## 4. Discussion

HAdV infection continues to be a serious cause of morbidity and mortality in populations at risk [3,5,12]. Though drugs capable of inhibiting HAdV replication are available, these are off-label uses and can result in significant toxicity to the patient [3,12]. Thus, there is a need for identification of compounds that can effectively and safely inhibit HAdV replication to treat infection.

Our results show that treatment with curcumin reduces both early and late gene expression (Figure 1), genome accumulation (Figure 3) and, ultimately, virus yield (Figure 4) for HAdV-5. Using late gene expression as a surrogate read-out for virus replicative capacity, we show that curcumin also inhibits HAdV-4 and HAdV-7 (Figure 6). The effects on early gene expression appear to be direct, as at 50 μM curcumin there was a significant reduction in E1A protein levels within treated cells (Figure 1B and Figure 7B) with no effect on cell health (Figure 2A and Figure 7E). However, extended exposure to 50 μM curcumin did reduce cellular metabolic activity by ~30% (Figure 2B andFigure 7F), suggesting at least part of the effect on late HAdV protein levels may be due to effects on cell health. In addition, E1A proteins are critically required for virus gene expression and replication [35], so it is possible that the later effects on virus function may also be due to an inability to generate sufficient quantities of E1A proteins. If the efficacy of curcumin is solely due to inhibition of E1A protein expression, forced expression of E1A should rescue the ability of HAdV to replicate in curcumin-treated cells. We previously described an HAdV vector in which high-level E1A expression is driven by the human cytomegalovirus (HCMV) immediate early enhancer/promoter [19], and attempted to circumvent the curcumin-induced block in HAdV replication. Treatment with curcumin dramatically lowered the levels of E1A produced from this virus and, as a result, HAdV replication was not rescued. However, since the HCMV immediate early enhancer/promoter is the first promoter activated during native HCMV infection and drives expression of proteins crucial for efficient initiation of the HCMV lifecycle [42], our observation suggests that curcumin could also be an effective treatment for HCMV.

There are a number of potential mechanisms by which curcumin may inhibit HAdV gene expression and replication. First, curcumin upregulates expression of the death-domain-associated protein (Daxx) [43], a protein found in promyelocytic leukemia protein (PML) nuclear bodies (PML-NB) that are involved in an interferon (IFN)-induced antiviral response against HAdV [44]. During infection, the HAdV proteins E4 ORF3 and E1B-55k normally antagonize this response [44,45]. Ineffective expression of early viral proteins caused by curcumin may prevent HAdV from shutting down this anti-viral pathway. Second, as previously reported by our research group, HDAC activity is required for HAdV replication [19], and curcumin has been reported to lower HDAC activity [46]. Finally, curcumin can inhibit the activity of p300/CREB-binding protein (p300/CBP) [47], a key cellular protein that interact with E1A and mediates global changes in gene expression within the cell to modify the microenvironment for optimal viral replication [48]. Any or all these mechanisms may be involved in the anti-HAdV effects of curcumin.

Our studies revealed that, although showing some efficacy against HAdV, curcumin displays a very narrow therapeutic window. Similar to our observations, previous work using curcumin against A549 cells as an in vitro cancer model showed that curcumin can cause apoptosis in A549 cells [49]. Thus, a balance must be reached between efficacy against HAdV and maintaining health of the cell. Curcumin may show greater efficacy as an anti-HAdV agent in non-transformed cell lines or patients. Curcumin also naturally shows poor bioavailability and stability at physiological pH [50], which would limit its distribution to infected tissues in vivo. However, many research groups have synthesized and evaluated curcumin derivatives that show enhanced bioavailability, stability, and/or anti-viral activity [30,34,51]. The use of nanoparticles and other formulations and carriers can enhance curcumin solubility and bioactivity [21], which can improve antiviral activity [52,53,54]. Such approaches may enhance efficacy of curcumin against HAdV.

In summary, our work shows that curcumin can reduce HAdV early and late gene expression, as well as virus yield, in vitro. Our work extends previous observations that curcumin is capable of inhibiting other viruses, including zika virus, human immunodeficiency virus, and influenza A virus, among many others [29]. Thus, curcumin-derivative compounds or formulations that reduce toxicity while increasing efficacy may find use as effective broad-spectrum antiviral therapeutics.
